# Brain MR Elastography Metrics Associated with Alterations in Learning and Memory in People with HIV

**DOI:** 10.21203/rs.3.rs-9828473/v1

**Published:** 2026-06-15

**Authors:** Abrar Faiyaz, Miriam Weber, Meera V Singh, Irteza E. Kabir, Kevin J. Parker, Ingolf Sack, Marvin M Doyley, Md Nasir Uddin, Giovanni Schifitto

**Affiliations:** University of Rochester; University of Rochester; University of Rochester; University of Rochester; University of Rochester; Charité – Universitätsmedizin Berlin; University of Rochester; University of Rochester; University of Rochester

**Keywords:** MRE, HIV, Learning, Memory, Elastography, Viscosity

## Abstract

High prevalence of HIV-associated neurocognitive impairment persists despite effective antiretroviral therapy. While conventional neuroimaging often fails to fully explain cognitive deficits in virally suppressed people with HIV (PWH), magnetic resonance elastography (MRE) offers a novel approach to quantify microstructural tissue coherence through brain biomechanics. This study investigated whether MRE-derived biomechanical metrics exhibit a pathological, group-specific relationship with cognitive performance in PWH, and compared their sensitivity against advanced diffusion MRI and peripheral biomarkers of neuronal injury and inflammation. In a cross-sectional cohort of 27 virally suppressed PWH and 30 matched healthy controls, participants underwent comprehensive cognitive testing, multi-frequency MRE (measuring stiffness, viscosity, and strain), multi-shell diffusion MRI (including NODDI metrics), and blood sampling for plasma neurofilament light chain and glial fibrillary acidic protein. PWH exhibited significantly lower cognitive scores than controls, particularly in learning and memory. Although baseline mean values across imaging and peripheral biomarkers did not notably differ between the groups, a significant group-by-cognition interaction emerged exclusively for MRE parameters. Specifically, in PWH, elevated viscosity and reduced strain correlated with poorer attention, whereas lower viscosity and strain correlated with better memory. These biomechanical-behavioral relationships were absent in healthy controls, and no notable interactions were observed for advanced diffusion metrics or blood biomarkers. Ultimately, brain biomechanics measured by MRE demonstrate greater sensitivity to altered cognitive function in PWH than advanced diffusion MRI and peripheral biomarkers, highlighting their potential as functional correlates of neurocognitive impairment that warrant further longitudinal validation.

## Introduction

Combined antiretroviral therapy (cART) has transformed HIV into a manageable condition, significantly extending life expectancy (1). However, 30–50% of PWH still experience HIV-associated neurocognitive impairment, characterized by deficits in learning, memory, and attention(2, 3). These impairments persist despite viral suppression, hindering daily functioning and medication adherence(4, 5). Sensitive in-vivo biomarkers are needed to detect the subtle neuropathology driving this chronic cognitive dysfunction.

In the modern cART era, HIV associated neuropathology is largely attributed to a state of chronic, low-grade neuroinflammation, mediated by glia activation and subsequent synaptic injury (6–9). Several neuroimaging modalities have documented structural brain injury in PWH, affecting gray and white matter, particularly using morphometry and diffusion-weighted imaging (10–13). Advanced diffusion MRI (dMRI) techniques, such as Neurite Orientation Dispersion and Density Imaging (NODDI) and tensor-valued diffusion MRI, have been employed to probe microstructural integrity in the context of inflammatory changes and the relationships with cognitive performance and blood markers (14–17).

Changes in brain stiffness, as measured by MRE, have been documented in several neurodegenerative disorders (18) and in inflammatory conditions such as multiple sclerosis (19, 20). By measuring tissue stiffness (elasticity, G), viscosity (fluid-like resistance, φ), and strain (degree of deformation), MRE provides information about the integrity of the cellular microenvironment and extracellular matrix(21, 22). Changes in these properties have been linked to the glial activation and synaptic alterations that characterize other neurodegenerative diseases(18).

Blood biomarkers of neuronal injury, plasma neurofilament light chain (NfL), and glial activation, glial fibrillary acidic protein (GFAP), are elevated in neurodegenerative disorders and neuroinflammatory conditions, including Alzheimer’s disease(23), multiple sclerosis (24, 25), and HIV (26).

In this study, we investigated the relationship between MRE metrics representing brain biomechanics via stiffness, viscosity, and strain, advanced diffusion MRI, peripheral biomarkers of injury and inflammation, and cognitive performance in a cohort of cART-treated PWH and healthy controls. We hypothesized that MRE metrics (tissue stiffness, viscosity, and strain) would differ between PWH and PWOH and be associated with cognitive function. We posited that MRE will provide significant complementary information to other imaging modalities in assessing HIV-associated brain injury.

## Methods

The experiments reported in the study were performed in accordance with relevant guidelines and regulations; and the details follows.

### Study Participants

The study was conducted in accordance with the institutional review board protocol, and all participants provided written informed consent prior to enrollment. An initial cohort of 30 individuals with HIV (PWH) and 36 healthy controls (HC) was recruited between 2019 to 2021. All PWH were on stable antiretroviral therapy with suppressed plasma viral loads. PWH and PWOH were excluded if they had a history of traumatic brain injury, loss of consciousness > 30 minutes due to any cause, history of brain infections (other than HIV for PWH), stroke, or other space-occupying lesions. Following image acquisition, MRE data underwent a quality control check for shear wave amplitude and propagation quality. Data from three PWH and six HCs were excluded due to insufficient wave penetration or imaging artifacts. The final analytic cohort, therefore, consisted of 27 PWH (mean age ± SE = 57.1 ± 2.0 years; 7 female) and 30 HC (mean age ± SE = 57.7 ± 2.7 years; 8 female).

### Image Acquisition Protocols

All MRI data were acquired on a 3T Siemens Prisma scanner (Erlangen, Germany) dedicated to research. The system was equipped with high-performance gradients (maximum strength 80 mT/m, slew rate 200 T/m/s) and utilized a 64-channel phased-array receive head coil for signal reception with body coil transmission.

### Anatomical Imaging

High-resolution structural images were obtained using a T1-weighted 3D Magnetization Prepared Rapid Gradient Echo (MPRAGE) sequence for anatomical reference and segmentation. Key acquisition parameters were repetition time (TR) = 1840 ms; echo time (TE) = 2.34 ms; inversion time (TI) = 962 ms; and an isotropic voxel size of 1.0 mm^3^.

### MR Elastography

Multifrequency MRE was performed to quantify brain tissue viscoelasticity. Harmonic shear waves were generated at three distinct frequencies (20, 40, and 50 Hz) by a pneumatic driver system. Wave propagation was captured using a motion-sensitized single-shot spin-echo echo-planar imaging (SE-EPI) sequence with the following parameters (Scan time-~5–6 minutes): TR/TE = 5100/76 ms; 2.0 mm^3^ isotropic resolution. To encode the three-dimensional tissue displacement, a motion-encoding gradient (MEG) with an amplitude of 60 mT/m and a frequency of 31.81 Hz was applied along three orthogonal directions. The resulting wave fields were sampled at eight phase offsets.

### Diffusion MRI

Multi-shell diffusion-weighted images (DWI) were acquired using a 2D SE-EPI sequence with simultaneous multi-slice acceleration. The protocol included two shells with b-values of 1000 and 2000 s/mm^2^, each with 64 diffusion-encoding directions, along with seven non-diffusion-weighted (b = 0) volumes. Additional parameters were TR/TE = 4300/69.0 ms and an isotropic resolution of 1.5 mm^3^. To correct for susceptibility-induced geometric distortions, the entire DWI acquisition was repeated with an opposite phase-encoding polarity.

### Clinical and Neuropsychological Assessment Fluid Biomarker Analysis

Peripheral blood (40 ml) was collected via venipuncture into ACD vacutainers and processed within two hours. Samples were maintained at room temperature with gentle agitation before centrifugation (10 minutes at 1000 x g) to isolate plasma. Aliquots were cryopreserved at −80°C. GFAP and NfL levels were measured via Quanterix Simoa 2-plex assays. All procedures followed manufacturer protocols.

### Neurocognitive Assessment

A comprehensive neuropsychological battery was administered to all participants to evaluate performance across seven distinct cognitive domains. Information processing speed was measured with the Symbol Digit Modalities Test, the Stroop Color and Word Test (Color Naming), and the Trailmaking Test A. Executive function was assessed using the Stroop Color and Word Test (Interference), and the Trailmaking Test B. Attention and working memory were evaluated via the Letter Number Sequencing subtest of the Weschsler Adult Intelligence Scale and the California Computerized Assessment Package (CalCAP). The learning domain included the Immediate recall trials of the Rey Auditory Verbal Learning Test and the Rey Complex Figure Test, while the memory domain utilized the delayed recall portions of both tests. Language was assessed with the Controlled Oral Word Association Test, and motor function was measured using the Grooved Pegboard test.

The tests were administered and scored by trained psychometrists under the supervision of a licensed clinical neuropsychologist. For each individual test, raw scores were converted to standardized z-scores using published, manualized norms that account for age and education. Composite domain scores were then computed by averaging the z-scores of the constituent tests within each of the seven domains. A total cognitive z-score was also calculated by summing the domain-specific z-scores.

### Image Processing and Analysis

All image processing was conducted using combination of standardized software packages, including FSL (v6.0.0), MRtrix3, Advanced Normalization Tools (ANTs) and custom-scripts in MATLAB-R2024.

### MRE Reconstruction

The multifrequency MRE data were processed using a multi-frequency dual elasto-visco (MDEV) inversion algorithm(27). This method calculates the complex shear modulus, G*=G′+iG′′, where G′ is the storage modulus and G′′ is the loss modulus. From this, two key viscoelastic parameters were derived:

1. **Magnitude of the complex shear modulus (**∣G*∣ **in kPa)**: While ∣G*∣ incorporates both storage (elastic) and loss (viscous) components, it is referred to here as **stiffness** for brevity, as G′≫G′′ in brain tissue.
2. **Phase angle (**ϕ **= arctan(G′′/G′) in radians)**: This parameter represents the **relative viscosity** (or damping ratio) of the tissue, indicating the weight of viscous relative to elastic properties(28).
3. **Strain**: Derived using the same inversion method to quantify tissue deformability.

The frequencies of 20, 40, and 50 Hz were selected to cover the typical range used in clinical MRE, providing a balance between wave penetration at lower frequencies and spatial resolution of mechanical properties at higher frequencies(29, 30).

### Diffusion MRI Processing

The raw DWI data were first preprocessed to correct for susceptibility-induced distortions, eddy currents, and subject motion using FSL’s topup and eddy tools(31). Following preprocessing, all dMRI and MRE metrics were calculated using tools within the MRtrix3 software package. Both DTI and NODDI models were fitted to the data to generate maps of fractional anisotropy (FA), and mean diffusivity (MD); neurite density index (NDI), orientation dispersion index (ODI), and the isotropic volume fraction (fISO) respectively.

### Region of Interest (ROI) Analysis

To facilitate robust regional analysis, all imaging data were co-registered to a common space for each subject. Specifically, the MRE parameter maps and standardized brain atlases (including Harvard-Oxford, and JHU-ICBM) were transformed into each subjec’s native diffusion space using non-linear registration with ANTs. Mean values for all MRE and dMRI metrics were then extracted from three global regions of interest: GM cortical, GM subcortical, WMTracts.

### Statistical Analysis

Statistical analyses were performed in MATLAB. Differences in demographic characteristics between groups were assessed using two-sample t-tests for continuous variables and chi-square tests for categorical variables. Group differences in cognitive domain scores were evaluated using an ANCOVA with age as a covariate.

The primary hypothesis was tested using a general linear model to assess the statistical interaction between group (PWH vs. HC) and cognitive scores in predicting imaging metrics. For each metric and cognitive score, we fitted the model:

MRI/MREMetric~Cognition+Group+Cognition*Group

The p-value for the interaction term was taken as the measure of a significant group-specific relationship. To account for multiple comparisons, we applied the Benjamini-Hochberg False Discovery Rate (FDR) correction to our primary family of 36 MRE-based interaction tests. A finding was considered statistically significant if its FDR-corrected p-value (q-value) was less than 0.05.

## Results

### Cohort Demographics and Cognitive Performance

The groups were well-matched for age, sex, ethnicity, and race (all p > 0.05). There was a trend toward lower educational attainment in the PWH group, but this did not reach statistical significance (p = 0.072).

**Figure 1:**
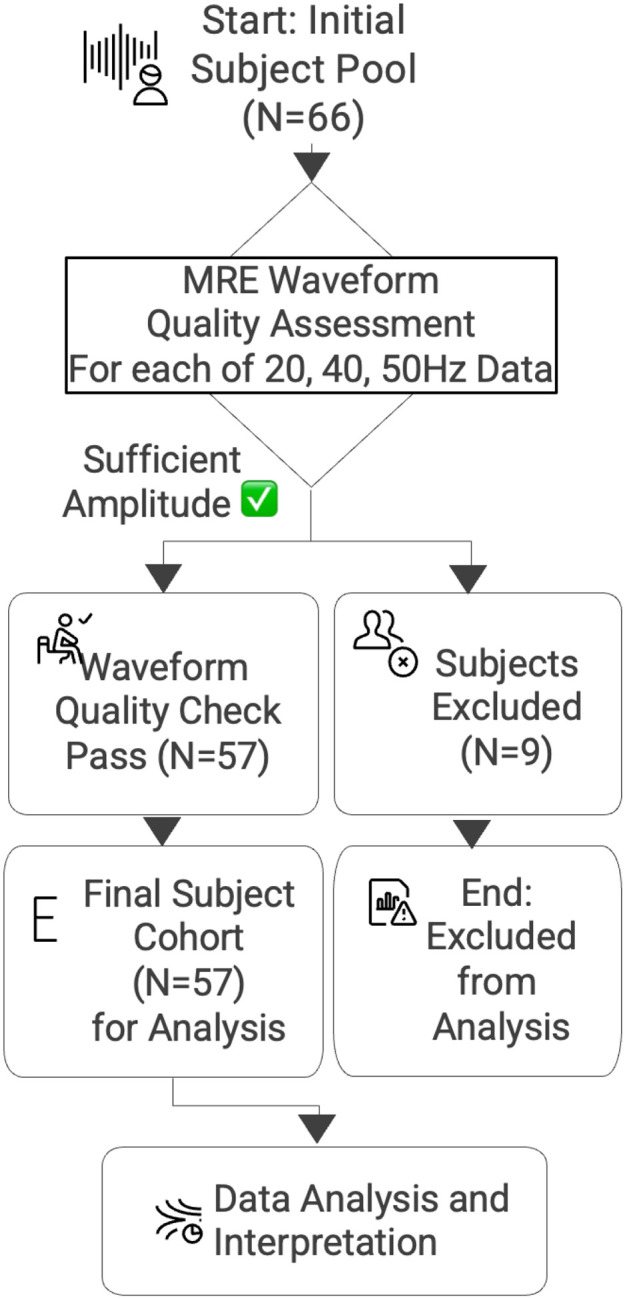
Flow diagram of participant exclusion due to inadequate amplitude of the oscillator paddle in all the frequencies.

A detailed summary of participant demographics is provided in Table 1 and Fig. 1.

Consistent with previous literature documenting persistent deficits in attention, learning, and memory among cART-treated PWH(2, 32), the PWH group demonstrated significantly poorer cognitive performance compared to healthy controls. After correcting for age, PWH had a significantly lower Total Cognitive Z-Score (p = 0.001, Cohen’s d = −0.91). Domain-specific analysis revealed that PWH performed worse on measures of Learning (p = 0.0002, d = −1.18), Memory (p = 0.006, d = −0.90), and Language (p = 0.026, d = −0.81). No significant group differences were observed for executive function, processing speed, attention, or motor skills.

### Group Comparisons of MRI/MRE metrics and Blood Biomarkers

**Figure 2:**
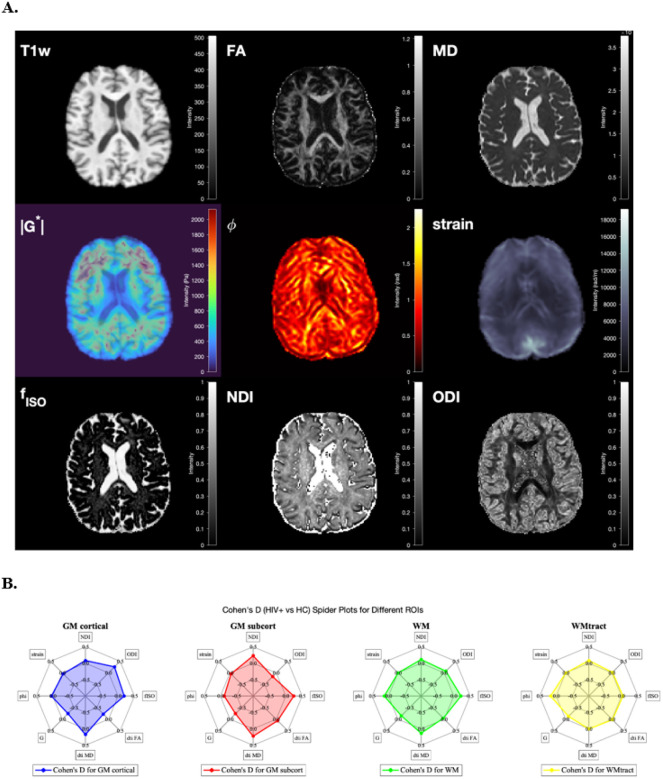
**A.** Representative multimodal brain images from a healthy subject. **B.** Effect size (Cohen’s d) demonstrating minimal group differences between the people living with HIV (PWH) and Healthy Control (HC). Small to very small effects were observed for viscosity (phi) in white matter (WM); stiffness G in GM (cortical and subcortical); fISO and ODI in cortical GM; and NDI, fISO, ODI, MD in subcortical GM.

We next assessed for group-level differences in the imaging and peripheral biomarker metrics themselves. There were no statistically significant differences between the PWH and HC groups in any MRE metric [stiffness (G), viscosity (φ), strain], diffusion MRI metrics (NDI, ODI, fISO, FA, MD), or peripheral biomarker (NfL, GFAP) and, Reynolds Risk Score across any of the brain regions analyzed. (See **Supplementary Table S1** for a full summary of group comparisons). Figure 2A. shows structural, diffusion and elastography related brain images subject to our study. Figure 2B. shows that the effect size (Cohen’s d) is minimal for the group difference between PWH and Healthy Control. Small effect is noted for viscosity (phi) in WM; stiffness G in GM (cortical and subcortical); fISO and ODI in cortical GM; and NDI, fISO, ODI, DTI-MD in Subcortical GM.

### MRE Metrics Reveal Significant Brain-Behavior Relationship in PWH

To test whether the relationship between brain tissue properties and cognition was different between groups, we performed a formal interaction analysis for each imaging and biomarker metric. This analysis revealed that MRE-derived viscosity and strain, but not stiffness, showed significant group interactions with specific cognitive domains. These findings remained significant after FDR correction for multiple comparisons (Table 2).

### Viscosity Interactions

**Figure 3:**
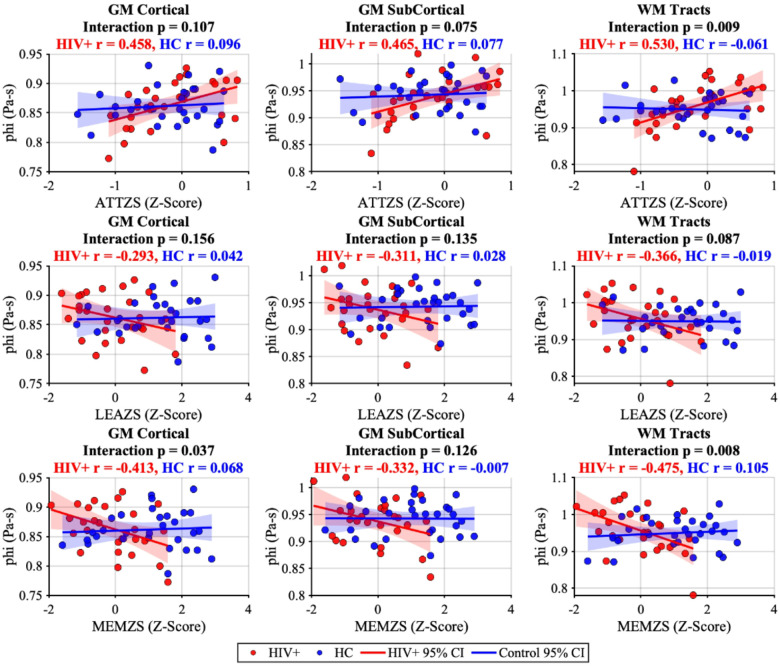
Group interactions and correlation coefficients for viscosity in people living with HIV (PWH) and healthy controls (HC).

A significant interaction was found between group and attention scores for viscosity in the white matter tracts (q = 0.044). Post hoc analysis of these interactions revealed that, in PWH, higher brain tissue viscosity was significantly associated with better attention performance. In contrast, no such relationship was observed in healthy controls. A similar significant interaction was found for memory, with the association between viscosity and cognition existing only in PWH (q = 0.044). However, in this analysis, higher viscosity in the white matter tracts was associated with worse memory scores. Figure 3 demonstrates the group interactions and reports the correlation coefficients of the PWH and HC in terms of viscosity.

### Strain Interactions

Consistent findings were observed for strain, where significant interactions with attention were present across: GM Cortical (q = 0.032), GM Subcortical (q = 0.032), WM (q = 0.032). In all cases, the interaction was driven by a significant negative association in the PWH group, where lower strain was linked to better attention scores. Conversely, this was absent in the control group.

**Figure 4:**
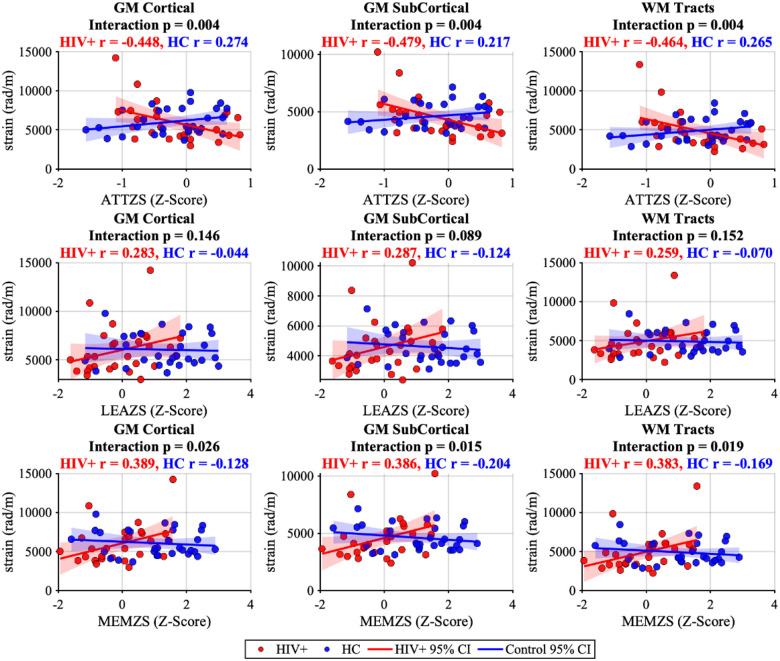
Group interactions and correlation coefficients for strain in PWH and HC.

We observed a significant interaction between strain and memory scores across all three ROIs at an uncorrected threshold (p < 0.05 for all). However, these findings did not reach significance after FDR correction. Interestingly, in contrast to the Attention domain, there was a positive association between strain and cognitive performance in PWH. Figure 4 demonstrates the group interactions and reports correlation coefficients for PWH and HC in terms of strain.

### Diffusion MRI vs. Peripheral Biomarkers Do Not Show Specific Interactions

In contrast to the MRE findings, no significant interaction effects were found for any of the advanced diffusion metrics (NDI, ODI, f_ISO_, FA, MD) or the peripheral biomarkers of neuronal injury (NfL), glial activation (GFAP) or vascular risk with any cognitive domain after correction for multiple comparisons. While several uncorrected trends were noted (e.g., between NfL and memory, p = 0.06), none approached the statistical significance or consistency of the MRE-based interactions. A full summary of all interaction p-values (FDR Corrected) is provided in **Supplementary Tables 2–13** and **Supplementary Figs. 1–2**.

## Discussion

Our study demonstrates that brain viscoelastic properties, as measured by MRE metrics, are associated with cognitive function in PWH. This suggests a possible pathomechanism for the persistent attention and memory deficits experienced by 30–50% of PWH (2, 26). Our primary finding reveals a consistent, group-specific link between MRE metrics and cognition, but this differed across cognitive domains. In PWH, increased viscosity and decreased strain—indicating reduced tissue deformability—correlated significantly with poorer memory but better attention performance. This relationship was absent in healthy controls. Interestingly, in our sample, PWH demonstrated worse memory function than healthy controls, but their attention performance did not differ from that of healthy controls. This behavioral dissociation frames our biomechanical findings, i.e., for memory, which is compromised, lower viscosity was associated with *better* performance. This suggests that elevated viscosity in HIV-associated WM Tract likely represents a pathological load—such as chronic gliosis or inflammatory cellular infiltration—that actively disrupts memory networks(27, 28). It is possible that the networks subserving the cognitive domains may not be simultaneously affected to the same extent. Therefore, networks affected by more acute inflammation will have different viscoelastic properties than those affected by chronic inflammation(28). Thus, MRE-derived viscosity appears to capture different stages of pathology. Of interest, our analysis revealed no significant group-specific relationships between cognitive performance and plasma levels of neuronal injury (NfL), glial activation (GFAP). However, NFL plasma levels were significantly inversely correlated with viscosity and positively correlated with strain (**Supplementary Fig. 1**).

The dMRI metrics (FA, MD, NDI, ODI, and fISO) showed no association with cognitive performance. These results suggest that mechanical microstructural changes captured by MRE may precede the alterations detectable by dMRI. While diffusion metrics reflect water displacement within cellular boundaries(13, 14), MRE probes the mechanical integrity of the extracellular matrix (ECM) and neuronal architecture (21, 22). The ECM is a complex network that regulates synaptic plasticity and stability (33). MRE viscosity is sensitive to ECM density and the friction of cell-matrix interactions(28, 30). Previous studies suggest that ECM remodeling and glial proliferation increase internal friction (viscosity) while restricting deformability (27, 29). Consequently, MRE may more directly measure synaptic and glial injury driving cognitive decline in this population (19, 22). However, we did not find a significant correlation between viscosity or strain and the glia marker GFAP (**supplementary Fig. 1**).

The sensitivity of ϕ (relative viscosity) to cognitive performance in PWH supports ‘damping’ as a marker of neural health. ϕ represents the tissue’s viscous-to-elastic balance, aligning with Johnson et al., who showed that the damping ratio—a mechanical analog to ϕ—predicts microstructural integrity and memory (34). While global stiffness reflects gross neurodegeneration, damping properties more closely track hippocampal microarchitecture density and connectivity, which are essential for memory (34, 35). Contextualizing these findings, MRE studies in Alzheimer’s (AD) and Multiple Sclerosis (MS) typically show global softening due to neuronal loss, demyelination, and ECM breakdown(18, 20). Normal aging similarly involves declining viscoelastic integrity(22). Our findings in the memory domain were consistent with these previous reports; lower viscoelastic integrity was associated with worse memory function.

Our study has limitations, including a cross-sectional design that prevents causal inference about whether biomechanical changes predict future decline. Firstly, lack of data on the duration of HIV infection and the need to investigate concurrent structural changes, such as parenchymal volume loss and microvascular disease, which will be addressed in a future study. Second, while we controlled for major comorbidities, the potential confounding effects of specific antiretroviral regimens on brain tissue stiffness could not be fully ruled out. Additionally, the sample size was modest, necessitating validation in larger cohorts. Finally, MRE resolution is limited compared to the microscopic scale of cellular pathology; however, its macroscopic sensitivity makes it a valuable bridge between pathology and clinical function.

Nonetheless, our investigation suggests that brain biomechanics, as measured by MRE, are a distinctively sensitive correlate of cognitive decline in the modern era of HIV that requires extensive longitudinal validation. MRE appears to detect a subtle, pathologically relevant brain-behavior relationship; thus, it may be a useful tool to integrate with other established markers such as advanced dMRI. Future longitudinal studies are needed to confirm these findings and explore the potential of MRE-derived viscosity and strain as prognostic biomarkers for monitoring HIV associated cognitive impairment progression and evaluating the efficacy of neuroprotective therapies.

## Conclusion

This is the first study to provide evidence that MR elastography is sensitive to the neuropathological substrates of cognitive alterations in PWH. We show that brain viscosity and strain exhibit a significant association with attention and memory performance, specifically in PWH, a brain-behavior relationship that is absent in healthy controls. These findings encourage further, larger, and longitudinal MRE-based studies to measure HIV-associated brain injury and its relation to cognitive impairment.

## Supplementary Files

This is a list of supplementary files associated with this preprint. Click to download.

- SupplementalFileforReviewv05252026.docx

## Figures and Tables

**Table 1:** Demographic and Clinical Characteristics of Study Participants

Characteristics	PWH (n = 27)	HC (n = 30)	p-value
**Age, mean (SE)**	57.11 (2.04)	57.70 (2.68)	0.862
**Sex, n (%)**			0.949
Female	7 (25.93%)	8 (26.67%)	
Male	20 (74.07%)	22 (73.33%)	
**Ethnicity, n (%)**			0.687
Hispanic or Latino	1 (3.70%)	2 (6.67%)	
Not Hispanic or Latino	25 (92.59%)	28 (93.33%)	
Other	1 (3.70%)	0	
**Race, n (%)**			0.301
White	20 (74.07%)	25 (83.33%)	
Black or African American	5 (18.52%)	5 (16.67%)	
Other	2 (7.41%)	0	
**Education, n (%)**			0.072
≤ 12 Years	9 (33.33%)	4 (13.33%)	
> 12 Years	18 (66.67%)	26 (86.67%)	
**Total Cognitive Score, mean (SE)**	−0.389 (3.9)	3.47 (4.55)	**0.001**
**Peripheral Marker of Brain Injury, mean (SE)**			
NFL (pg/ml)	10.20 (8.53)	7.21 (4.52)	0.195
GFAP (pg/ml)	77.21 (48.78)	66.57 (36.98)	0.446
**Vascular Markers, mean (SE)**			
Reynolds Risk Score	6.96 (4.35)	7.27 (6.54)	0.836

**Table 2:** Significant MRE-Cognition Interaction Effects after FDR Correction Relationships between MRE and cognitive domain scores and their interaction tests that remained statistically significant (q < 0.05) after applying the Benjamini-Hochberg False Discovery Rate (FDR) correction across the 36 a priori MRE-cognition comparisons.

MRE Metrics	Interaction with Cognitive Domain (ROI)	Uncorrected p-value	FDR Corrected p-value (q)
**Viscosity**	Attention (WM)	0.001	0.032
	Attention (WM Tracts)	0.009	0.044
	Memory (WM Tracts)	0.008	0.044
**Strain**	Attention (GM Cortical)	0.004	0.032
	Attention (GM Subcortical)	0.004	0.032
	Attention (WM)	0.003	0.032
	Attention (WM Tracts)	0.004	0.032

GM: Gray Matter; WM: White Matter

## Data Availability

Anonymized data will be made available on reasonable request, pending appropriate institutional review board approvals.

## Footnote/Nonstandard Abbreviation and Acronyms:

cART: combined antiretroviral therapy
dMRI: diffusion magnetic resonance imaging
DTI: diffusion tensor imaging
ECM: extracellular matrix
FDR: false discovery rate
ϕ (phi): phase angle (relative viscosity)
G*: complex shear modulus
G′: storage modulus (elasticity)
G′′: loss modulus (viscosity)
GFAP: glial fibrillary acidic protein
MDEV: multi-frequency dual elasto-visco inversion
MRE: MR elastography
NDI: neurite density index
NfL: neurofilament light
NODDI: neurite orientation dispersion and density imaging
ODI: orientation dispersion index
PWH: people with HIV
